# Supplementary material for: Epigenetic age-predictor for mice based on three CpG sites
Source: eLife. 2018 Aug 24;7:e37462. doi: 10.7554/eLife.37462 (PMC6156076; doi:10.7554/eLife.37462)
Supplement: Supplementary file 1. [file elife-37462-supp1.docx]

## Supplementary File 1. Age-associated DNAm in nine genomic regions of the training set.

| **Gene name** | **Chromosome** | **CpG#** | **Position** | **R^2^** | **Slope of correlation** |
| --- | --- | --- | --- | --- | --- |
| *Prima1* | chr12 | **1** | **103214639** | **0,71** | **Negative** |
|  |  | 2 | 103214653 | 0,35 | Negative |
|  |  | 3 | 103214656 | 0,62 | Negative |
| *Hsf4* | chr8 | 1 | 105270965 | 0,62 | Positive |
|  |  | 2 | 105270996 | 0,81 | Positive |
|  |  | **3** | **105271000** | **0,95** | **Positive** |
|  |  | 4 | 105271006 | 0,91 | Positive |
|  |  | 5 | 105271015 | 0,90 | Positive |
|  |  | 6 | 105271021 | 0,87 | Positive |
|  |  | 7 | 105271025 | 0,81 | Positive |
| *Kcns1* | chr2 | 1 | 164168088 | 0,81 | Positive |
|  |  | **2** | **164168110** | **0,83** | **Positive** |
|  |  | 3 | 164168113 | 0,84 | Positive |
|  |  | 4 | 164168121 | 0,74 | Positive |
|  |  | 5 | 164168126 | 0,73 | Positive |
|  |  | 6 | 164168129 | 0,79 | Positive |
|  |  | 7 | 164168131 | 0,81 | Positive |
|  |  | 8 | 164168144 | 0,64 | Positive |
|  |  | 9 | 164168149 | 0,64 | Positive |
|  |  | 10 | 164168154 | 0,02 | Positive |
|  |  | 11 | 164168156 | 0,62 | Positive |
|  |  | 12 | 164168172 | 0,74 | Positive |
|  |  | 13 | 164168175 | 0,82 | Positive |
|  |  | 14 | 164168178 | 0,81 | Positive |
|  |  | 15 | 164168181 | 0,64 | Positive |
|  |  | 16 | 164168188 | 0,56 | Positive |
| *Gm9312* | chr12 | 1 | 24252012 | 0,37 | Positive |
|  |  | 2 | 24252014 | 0,47 | Positive |
|  |  | 3 | 24252028 | 0,14 | Positive |
|  |  | 4 | 24252032 | 0,21 | Positive |
|  |  | 5 | 24252037 | 0,45 | Positive |
|  |  | 6 | 24252044 | 0,43 | Positive |
|  |  | 7 | 24252050 | 0,25 | Positive |
|  |  | 8 | 24252062 | 0,27 | Positive |
|  |  | 9 | 24252069 | 0,53 | Positive |
|  |  | 10 | 24252073 | 0,15 | Positive |
|  |  | 11 | 24252075 | 0,48 | Positive |
|  |  | 12 | 24252082 | 0,16 | Positive |
|  |  | 13 | 24252092 | 0,53 | Positive |
|  |  | 14 | 24252095 | 0,50 | Positive |
|  |  | 15 | 24252097 | 0,55 | Positive |
| *Zfp148* | chr16 | 1 | 33382297 | 0,00 | Positive |
|  |  | 2 | 33382305 | 0,00 | Positive |
|  |  | 3 | 33382332 | 0,00 | Positive |
|  |  | 4 | 33382320 | 0,06 | Positive |
| *Prdm1* | chr10 | 1 | 44528354 | 0,01 | Positive |
|  |  | 2 | 44528381 | 0,22 | Positive |
|  |  | 3 | 44528389 | 0,18 | Positive |
|  |  | 4 | 44528401 | 0,21 | Positive |
| *Argap9* | chr10 | 1 | 127326467 | 0,00 | Negative |
|  |  | 2 | 127326473 | 0,08 | Negative |
|  |  | 3 | 127326477 | 0,02 | Negative |
|  |  | 4 | 127326483 | 0,03 | Negative |
|  |  | 5 | 127326488 | 0,03 | Negative |
|  |  | 6 | 127326514 | 0,01 | Negative |
|  |  | 7 | 127326525 | 0,29 | Negative |
|  |  | 8 | 127326529 | 0,11 | Negative |
|  |  | 9 | 127326603 | 0,25 | Negative |
| *Gm7325* | chr17 | 1 | 45601556 | 0,06 | Negative |
|  |  | 2 | 45601559 | 0,00 | Negative |
|  |  | 3 | 45601568 | 0,37 | Negative |
|  |  | 4 | 45601571 | 0,06 | Negative |
|  |  | 5 | 45601595 | 0,05 | Negative |
|  |  | 6 | 45601604 | 0,00 | Negative |
|  |  | 7 | 45601625 | 0,08 | Negative |
| *Mbd2* | chr18 | 1 | 70582482 | 0,00 | Positive |
|  |  | 2 | 70582500 | 0,11 | Positive |
|  |  | 3 | 70582508 | 0,22 | Negative |
|  |  | 4 | 70582512 | 0,20 | Positive |
|  |  | 5 | 70582514 | 0,04 | Positive |
|  |  | 6 | 70582523 | 0,01 | Positive |

CpG#: consecutive numbering of CpGs within each amplicon; Position refers to location on the corresponding chromosome (murine genome annotation: GRCm.38); R^2^ is the squared Pearson correlation of DNAm *versus* chronological age (pyrosequencing results of training set). Those CpGs that were subsequently implemented into the epigenetic aging model are highlighted in bold.
