# Supplementary material for: Epigenetic age-predictor for mice based on three CpG sites
Source: eLife. 2018 Aug 24;7:e37462. doi: 10.7554/eLife.37462 (PMC6156076; doi:10.7554/eLife.37462)
Supplement: Supplementary file 2. [file elife-37462-supp2.docx]

## Supplementary File 2. Multivariable model based on 15 CpGs

| **Gene name** | **Chromosome** | **CpG#** | **Position** | **R^2^** | **Coefficients** |
| --- | --- | --- | --- | --- | --- |
| (Intercept) |  |  |  |  | -1.62345 |
| *Prima1* | chr12 | 3 | 103214656 | 0,62 | -0.29869 |
| *Hsf4* | chr8 | 3 | 105271000 | 0,95 | 1.254757 |
| *Hsf4* | chr8 | 4 | 105271006 | 0,91 | 0.327821 |
| *Hsf4* | chr8 | 5 | 105271015 | 0,90 | 0.467297 |
| *Hsf4* | chr8 | 7 | 105271025 | 0,81 | 0.001369 |
| *Kcns1* | chr2 | 1 | 164168088 | 0,81 | 0.170725 |
| *Kcns1* | chr2 | 3 | 164168113 | 0,84 | 0.089469 |
| *Kcns1* | chr2 | 4 | 164168121 | 0,74 | 0.320572 |
| *Kcns1* | chr2 | 5 | 164168126 | 0,73 | 0.343513 |
| *Kcns1* | chr2 | 6 | 164168129 | 0,79 | 0.05701 |
| *Kcns1* | chr2 | 13 | 164168175 | 0,82 | 0.326642 |
| *Kcns1* | chr2 | 14 | 164168178 | 0,81 | 0.307202 |
| *Gm9312* | chr12 | 2 | 24252014 | 0,47 | 0.055652 |
| *Gm9312* | chr12 | 7 | 24252050 | 0,25 | 0.434457 |
| *Gm7325* | chr17 | 3 | 45601568 | 0,37 | -0.13861 |

This model was trained with a machine learning approach (linear model with L1 regularization; lambda = 1.570242). However, it comprised CpGs on five different amplicons and necessitates relatively long pyrosequencing reads. Therefore, it was not further validated.
