# Supplementary material for: Epigenetic age-predictor for mice based on three CpG sites
Source: eLife. 2018 Aug 24;7:e37462. doi: 10.7554/eLife.37462 (PMC6156076; doi:10.7554/eLife.37462)
Supplement: Supplementary file 3. [file elife-37462-supp3.docx]

## Table S3. Primers for pyrosequencing.

| **Primer** | **Sequence** |
| --- | --- |
| ***Prima1*** |  |
| Forward | 5'-TTGTGTTTAATTAGGAGAGGTAAATTATGAATTAGGTTTATA-3' |
| Reverse | 5'-Biotin-CAAAATTAATTACACCAACTTATAACCTACTATTC-3' |
| Sequencing | 5'-AATTATGAATTAGGTTTATATTT-3' |
| ***Hsf4*** |  |
| Forward | 5'-GTGAGTAGTAAGGTGGGATAAATTGTAGAAAAAATG-3' |
| Reverse | 5'-Biotin-TCCCTACTCTCCTACACTCCTCTCAAAACTTA-3' |
| Sequencing | 5'-ATTGTAGAAAAAATGGGAA-3' |
| ***Kcns1*** |  |
| Forward | 5'-GGTTGAGAGGGTGGTAGAAGAAGTTG-3' |
| Reverse | 5'-Biotin-ACTCCCCTCCATCCCTACCATATACATCCA-3' |
| Sequencing | 5'-GAAGATATTTAGAAGTTGAATT-3' |
| ***Gm9312*** |  |
| Forward | 5'-Biotin-TTGTTTTGGGGTATTAGAAATTTTTTT-3' |
| Reverse | 5'-CCTAACCATACTAAACCAAATCTCTATATCTAAAT-3' |
| Sequencing | 5'-AACCCCCACCACCTCTAATTTCAC-3' |
| ***Zfp148*** |  |
| Forward | 5'-Biotin-TTGGTGTATTTTAGTTTGGTTTTTGAAGGT-3' |
| Reverse | 5'-TCACTTCAAATTTCCTTAAACATTACAACCACTCCTA-3' |
| Sequencing | 5'-TTTTCTTTAATAACATCATA-3' |
| ***Prdm1*** |  |
| Forward | 5'-AATGAATGTAGTAGGAGGAATGAAGTAATAGGAGGTTT-3' |
| Reverse | 5'-Biotin-AAATCCTCCAAAACTACCCCAACTACTCAAATATAC-3' |
| Sequencing | 5'-TATTTTTTAAGGTAGTAAGAGT-3' |
| ***Arhgap9(1)*** |  |
| Forward | 5'-GGAAATAAAATTTTTTGTTTTAGTTTTTTTTATAATTGT-3' |
| Reverse | 5'-Biotin-ATCTCCTCCTCCCACTACAACAAAAACTCAT-3' |
| Sequencing | 5'-TTTTTAAAGATAGGGTGAT-3' |
| ***Arhgap9(2)*** |  |
| Forward | 5'-Biotin-GTAAGGTTTAGGGATATGAGTTTTTGTTGTAGT-3' |
| Reverse | 5'-CCTCCCCAATACCTTCCAAAATCT-3' |
| Sequencing | 5'-TTCCAAAATCTACACCC-3' |
| ***Gm7325*** |  |
| Forward | 5'-TGTTGGTTGAGGATAAAGAGTAGATAGTTTAGTAGAGT-3' |
| Reverse | 5'-Biotin-TTCCCTTTACAAATACAAATCCTACCATA-3' |
| Sequencing | 5'-TTTATGTTTTGGGAGTTTA-3' |
| ***Mbd2*** |  |
| Forward | 5'-TTATTAAGAAGTAGATATTAATTGGTTTTTAGTTTGTGTGTAT-3' |
| Reverse | 5'-Biotin-AAACCCCAACTAAAACCATCACCAAATCAA-3' |
| Sequencing | 5'-TAAGAAGTAGATATTAATTGG-3' |
